# Antibiofilm, Antimicrobial, and Anti‐inflammatory Potential of *Rubus chamaemorus* Rhizome, Leaf, and Fruit Extracts

**DOI:** 10.1002/cbdv.71479

**Published:** 2026-07-09

**Authors:** Hildegard Kieninger, Stefanie Koenig, Nadin Schultze, Ulrike Garscha, Sebastian Guenther, Christian Schulze

**Affiliations:** ^1^ Department of Pharmaceutical Biology Institute of Pharmacy Partner in the Greifswald Mire Center University of Greifswald Greifswald Germany; ^2^ Department of Pharmaceutical/Medicinal Chemistry Institute of Pharmacy University of Greifswald Greifswald Germany

**Keywords:** antimicrobial, cloudberry, leukotriene, natural products, virulence factor

## Abstract

The global rise of antimicrobial resistance is concomitant with an increasing prevalence of chronic wound infections. Medicinal plants are a promising source of novel therapeutic agents. *Rubus chamaemorus*, a plant native to Northern Europe traditionally used for wound healing, is one of them. Dried, powdered rhizomes, leaves, and fruits of *R. chamaemorus* were extracted with dichloromethane, methanol, and water. The extracts were screened for antibacterial and antifungal activity. Main compounds were identified using liquid chromatography‐mass spectrometry (LC‐MS). Biofilm inhibition was assessed using multidrug‐resistant *Escherichia coli*. Cytotoxicity against HaCaT cells, inhibition of 5‐lipoxygenase (5‐LOX) product formation, chelating, and antioxidant properties were determined. While the rhizome and fruit extracts demonstrated no relevant biological activities, the methanolic leaf extract exhibited a low minimal biofilm inhibitory concentration (MBIC = 30 µg/mL) and a potent 5‐LOX inhibition (IC_50_ = 1.5 ± 0.5 µg/mL). These properties are likely linked to the pronounced chelating and antioxidant properties of the extract. No cytotoxicity was observed in HaCaT cells and human primary monocytes (up to 200 and 30 µg/mL, respectively). LC‐MS analysis of the leaf extracts identified flavonoid glycosides as predominant compounds. These findings support the plant's traditional use in wound healing. Bioactivity‐guided fractionation is imperative to identify and characterize the active compounds.

## Graphical abstract

Rhizome, leaf, and fruit extracts of cloudberry (*Rubus chamaemorus* L., Rosaceae) are systematically evaluated for biological activities relevant to wound infections. The methanolic leaf extract exhibits potent antibiofilm and anti‐inflammatory activity without cytotoxicity, whereas rhizome and fruit extracts are largely inactive. LC‐MS analysis identifies flavonoid glycosides as the predominant constituents of the active leaf extract.

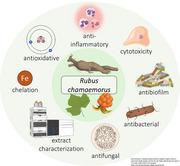

## 1 Introduction

Chronic wounds affect up to 2% of the population in developed countries annually [1, 2]. They are defined as wounds that fail to heal within three months and do not progress through the normal stages of wound healing, which are hemostasis, inflammation, proliferation, and tissue remodeling [3]. A prolonged inflammatory phase, driven by elevated levels of reactive oxygen species (ROS) and other pro‐inflammatory mediators, is a hallmark of chronic wounds [4, 5]. This sustained inflammatory response is often amplified by secondary bacterial infection, impaired tissue perfusion, ischemia, presence of necrotic debris, or underlying conditions such as diabetes mellitus [6]. Biofilm formation has been identified in up to 80% of chronic wounds, where its presence further impedes healing and tissue repair [7].

The predominant bacterial species with chronic wound‐associated biofilm formation include *Enterococcus faecium*, *Staphylococcus aureus*, *Klebsiella pneumoniae*, *Acinetobacter baumannii*, *Pseudomonas aeruginosa*, and *Enterobacter spp*., which are referred to as ESKAPE pathogens [8, 9]. According to the World Health Organization (WHO) 2024 Bacterial Priority Pathogens List, carbapenem‐resistant *A. baumannii*, *P. aeruginosa*, and *Enterobacterales*, as well as methicillin‐resistant *S. aureus* (MRSA), are particularly implicated in severe infections [10].

Antibiotic resistance and biofilm persistence substantially limit therapeutic options for chronic wound infections. Nevertheless, natural products represent a considerable pharmacological reservoir, with approximately 75% of existing drugs being derived from plant secondary metabolites [11]. Species of the genus Rubus are traditionally associated with beneficial effects on wound healing. However, only a limited number of studies have investigated the biological activity of *Rubus chamaemorus* fruits, and even fewer have examined other organs, such as rhizomes and leaves. In this context, *R. chamaemorus* L. (Rosaceae) could be a promising botanical source of novel secondary metabolites with anti‐inflammatory and antimicrobial properties.

*R. chamaemorus*, commonly known as cloudberry, is a perennial dioecious plant native to bogs in Northern Europe, Russia, and North America. The diverse medicinal applications of *R. chamaemorus* encompass all parts of the plant, including its fruits, the most extensively studied component, leaves, and rhizomes [12, 13, 14]. In summer, its female blossoms ripen into aggregate fruits, which were historically used as an antiscorbutic due to their high vitamin C content [12]. In addition to vitamin C, the fruits are also rich in vitamin E and tannins such as sanguiin‐H6 and lambertianin C [15]. Fermented cloudberry seeds have been shown to affect quorum sensing in *Chromobacterium violaceum* [16]. High concentrations (8 mg/mL) of aqueous and ethanolic seed extracts reduced biofilm formation and antibacterial activity against MRSA [17]. Acetone extracts of cloudberries have demonstrated antioxidative properties and antibacterial activity against *S. aureus* [18, 19]. Furthermore, administration of powdered fruits has been shown to protect against the development of metabolic inflammation in mice [20].

The use of rhizomes and leaves is mainly documented in traditional contexts, with the leaves employed to treat diarrhea and promote wound healing [13]. The antidiarrheal effects are attributed to the tannin content, as observed in other species within the genus *Rubus* [21]. The main components of cloudberry leaves are 4‐*O*‐α‐*L*‐arabinofuranosylellagic acid, quercetin 3‐*O*‐β‐*D*‐glucuronide, quercetin 3‐*O*‐β‐*D*‐2″‐galloylglucuronide, and kaempferol 3‐*O*‐β‐*D*‐glucuronide [22]. Regarding the biological activity of the leaves, mainly antimicrobial and biofilm inhibitory activity have been reported [23, 24]. In traditional medicine, the rhizomes of *R. chamaemorus* have been used to treat cough, fever, consumption, and to ease childbirth [14]. The absence of mycorrhiza, associated with the limited success of the inoculation with fungal spores, could indicate antifungal activity [25, 26].

Although the biological activity of cloudberry fruits has been well studied, the bioactivity of other plant parts remains comparatively undercharacterized. This study provides a comprehensive overview of the antibiofilm, antibacterial, and antifungal activities of all plant parts of *R. chamaemorus*. In particular, it examines properties relevant to traditional wound healing applications, such as anti‐inflammatory activity and keratinocyte cytotoxicity.

## 2 Results and Discussion

### 2.1 Evaluation of Antibacterial and Antifungal Activity

The antimicrobial activity of extracts from all parts of cloudberry was assessed. A panel of bacterial and fungal strains were screened using an agar disk diffusion assay. The selection included clinically relevant ESKAPE pathogens, as these are commonly associated with chronic wound biofilms and exhibit increasing prevalence in nosocomial infections [8, 9]. Fungal strains were selected based on the WHO fungal priority pathogens list, reflecting the rising concern over resistance to antifungal agents [27].

Inhibition zones were observed for several aqueous and methanolic extracts, irrespective of the plant part, particularly against two strains: *A. baumannii* and *S. aureus* (Table 1). No inhibitory activity was detected against the tested strains of *E. coli, P. aeruginosa, K. pneumoniae, E. faecalis, E. faecium, S. pyogenes, S. epidermidis, C. albicans, A. fumigatus, C. tropicalis*, and *M. circinelloides* (data not shown). The absence of antifungal activity of the rhizome extracts suggests that the lack of mycorrhiza is not a marker for antifungal defense compounds, as hypothesized by Nilsen et al. [25]. The disk diffusion assay confirms the previously reported higher susceptibility of *S. aureus* to the cloudberry leaf and fruit extracts than *C. albicans* and *E. coli* [24, 28]. The greater susceptibility of *S. aureus* compared to *P. aeruginosa* and *E. coli* is consistent with previous reports on the antibacterial effects of cloudberry seed extracts [29]. As with findings for *Rubus idaeus*, the fruits and leaves exhibited larger inhibition zones than the underground plant parts [30].

**TABLE 1:** Strains with an observed inhibition zone, ‐: no inhibition zone observed, Ø disk: 6 mm, mean ± standard deviation [mm], *n* = 3.

	Rhizome			Leaf			Fruit		
	DCM	MeOH	H_2_O	DCM	MeOH	H_2_O	DCM	MeOH	H_2_O
*S. aureus* PBIO483	—	9.0 ± 0.0	—	—	10.3 ± 1.5	8.7 ± 1.5	—	10.0 ± 1.0	7.3 ± 0.6
*S. aureus* PBIO901	—	8.3 ± 0.6	—	—	8.0 ± 6.9	8.3 ± 0.6	—	9.3 ± 1.5	5.3 ± 4.6
*A. baumannii* PBIO2202	—	—	—	—	11.3 ± 1.2	—	—	—	—
*A. baumannii* PBIO2212	—	—	—	—	12.7 ± 2.1	—	—	—	—

A microdilution assay was used to assess the susceptibility of the sensitive strains. Of the extracts tested, only the methanolic leaf extract inhibited the growth of *A. baumannii* PBIO2212 at a concentration of 1 mg/mL when determined visually. However, the high concentration required to achieve this effect renders cloudberry extracts negligible for antibacterial applications.

### 2.2 Biofilm Inhibition

A key virulence factor of multidrug‐resistant strains is their capacity to form biofilms [31, 32]. They encapsulate the bacteria with a matrix of polysaccharides and proteins acting as a diffusion barrier that enhances resistance to both physical and chemical stimuli [33]. The eradication of biofilm‐forming pathogens often requires antibiotic concentrations that are up to 1000 times higher than those needed to eliminate them in their planktonic state [34].

**FIGURE 1:**
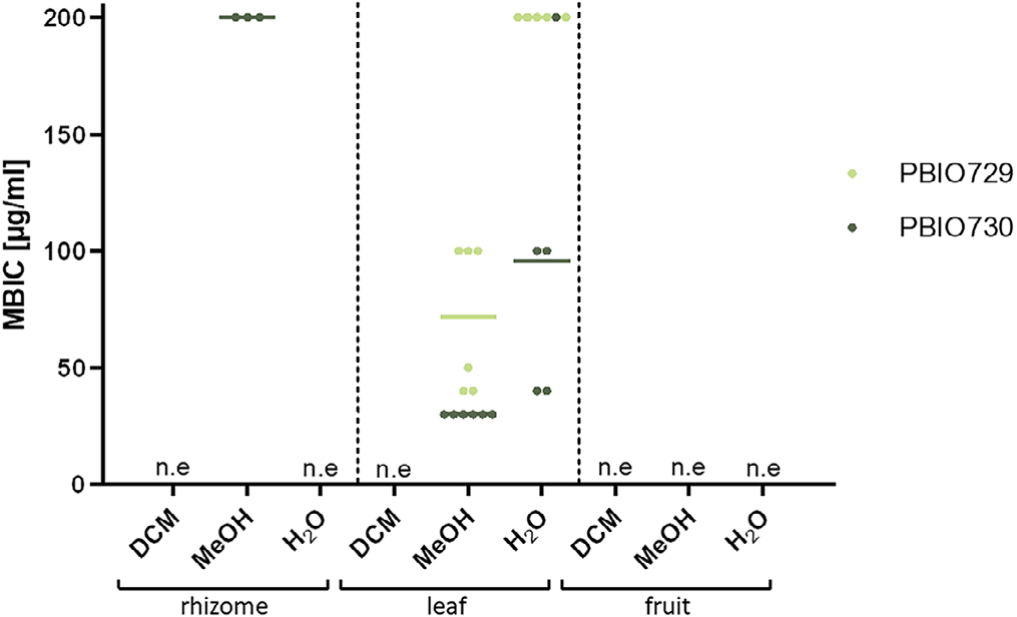
Minimal biofilm inhibitory concentration (MBIC) of cloudberry extracts against two *E. coli* strains (PBIO729 and PBIO730), n.e.: no effect at highest tested concentration of 200 µg/mL; dots represent individual experiments, line represents the mean of each strain.

The influence of the rhizome, leaf, and fruit extracts on the formation of two extracellular matrix components was evaluated with a phenotypic assay. Therefore, two multidrug‐resistant *E. coli* strains (PBIO729 and PBIO730) with a high expression of these components, curli and cellulose, were selected. Discoloration and a shiny surface indicate inhibition of curli and cellulose formation. The minimum biofilm inhibitory concentration (MBIC) was assessed visually (Figure 1). Small variations in activity are therefore not measurable. Among the rhizome extracts, biofilm inhibition was observed only for the methanolic extract at 200 µg/mL, and exclusively in the strain PBIO730. None of the fruit extracts exhibited any biofilm inhibitory activity. The strongest biofilm inhibition was observed for PBIO730 and the methanolic leaf extract at 30 µg/mL (Figure 1), confirming the results of Neumann et al. [23]. For PBIO729, the methanolic leaf extract displayed an MBIC of 72 µg/mL. The aqueous leaf extract showed an MBIC of 100 µg/mL for PBIO730 and 200 µg/mL for PBIO729. PBIO729 required higher concentrations for biofilm inhibition and therefore seems to be less susceptible than PBIO730. These findings are consistent with those of Neumann et al., but contrast with observations with sundew extracts, where PBIO729 was more sensitive than PBIO730 [23, 35].

To test for antimicrobial activity, the extracts were examined at concentrations ranging from 0.1 to 1 mg/mL. The antibacterial activity of all tested extracts against PBIO729 and PBIO730 was determined visually in liquid culture and is above 1 mg/mL for the leaf, fruit, and rhizome extracts. Thus, the biofilm inhibition is not caused by growth inhibition, but through an interference in the biofilm forming pathway.

### 2.3 Evaluation of Cytotoxic Activity

**FIGURE 2:**
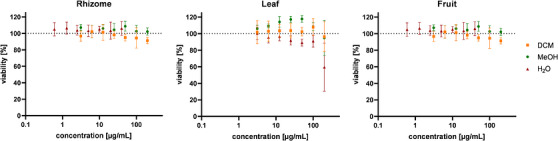
Cell viability of HaCaT cells treated with *R. chamaemorus* rhizome, leaf, and fruit extracts for 24 h (MTT‐Assay). The values were referred to solvent‐treated cells. Dotted line represents 100% viability of solvent treated cells. Mean ± standard deviation of *n* = 3 with 3 technical replicates.

Given the traditional use of leaves from the genus *Rubus* for their wound healing properties, the safety of topical application was assessed by evaluating cytotoxicity in HaCaT cells, a keratinocyte model widely used in dermatological research [36]. None of the rhizome, leaf, or fruit extracts reduced the cell viability below 50% at concentrations up to 200 µg/mL. For the aqueous rhizome and fruit extracts, concentrations were tested up to 40 µg/mL (Figure 2). The results are comparable for *Rubus parvifolius* leaves, a plant from the same genus with a similar phytochemical profile [37]. The absence of cytotoxicity *in vitro* suggests that the cloudberry extracts are potentially suitable for topical application.

### 2.4 Anti‐Inflammatory Properties

Polymorphonuclear leukocytes (PMNLs) are a subset of white blood cells comprising neutrophils, eosinophils, and basophils. Beyond their role in phagocytosis and pathogen defense via enzyme‐rich granules, PMNLs migrate to sites of infection or injury, mediating inflammation and tissue repair. A key mediator in these processes is 5‐LOX, an enzyme central to the biosynthesis of pro‐inflammatory leukotrienes from the polyunsaturated fatty acid arachidonic acid. Inhibiting 5‐LOX activity reduces leukotriene production, thereby attenuating inflammatory responses [38].

**FIGURE 3:**
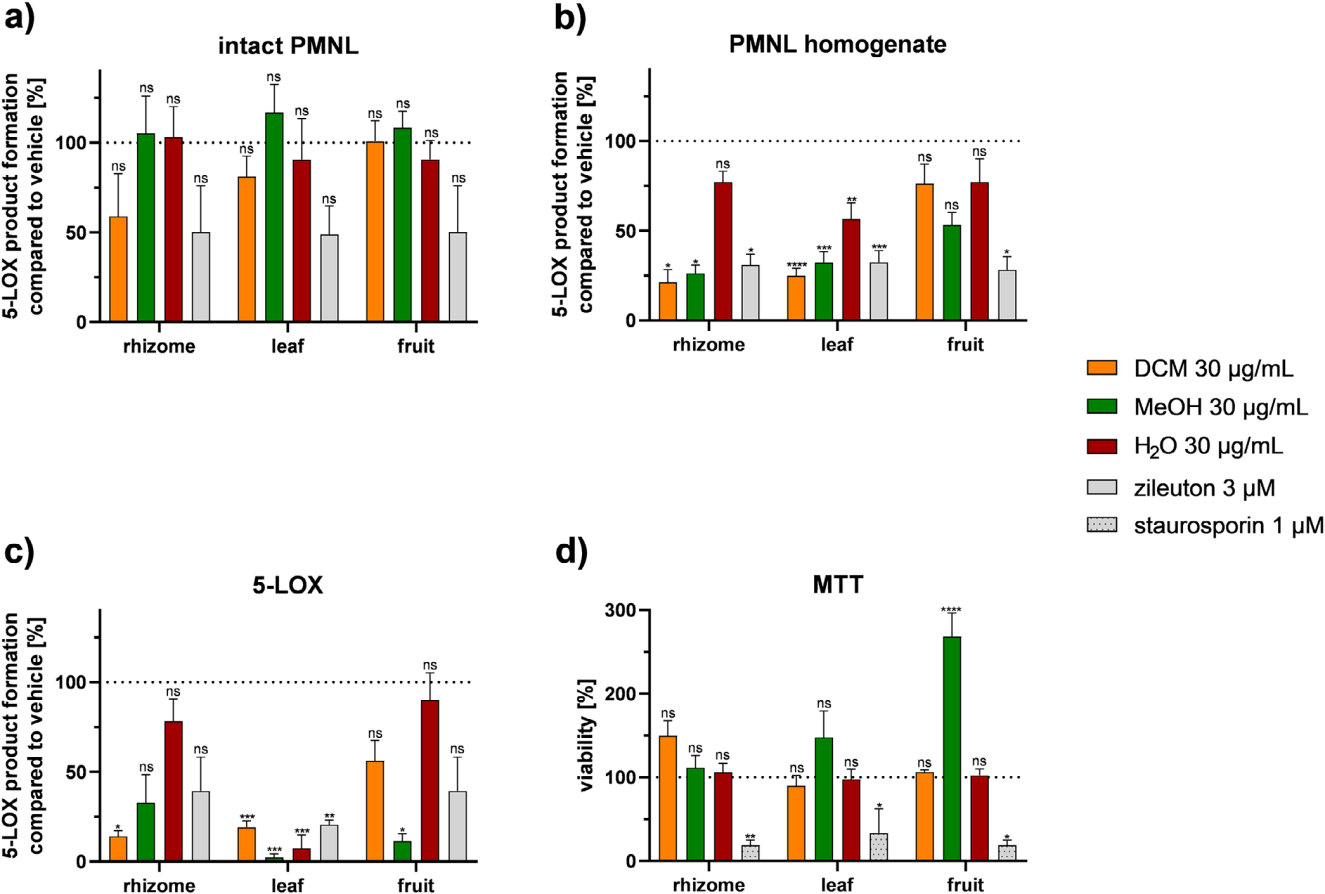
(a–c) Effects of DCM, MeOH, and H2O rhizome, leaf, and fruit extracts on intact PMNLs, PMNL homogenate, and purified recombinant 5‐LOX regarding 5‐LOX product formation; zileuton as positive control; dotted line represents 100% 5‐LOX product formation of vehicle; mean + standard deviation; *n* = 3‐4. (d) MTT assay assessing monocyte viability after treatment with rhizome, leaf, and fruit extracts (24 h); staurosporin as positive control; dotted line represents 100% viability of solvent treated cells; mean + standard deviation; *n* = 3‐4. Statistical analysis performed with One‐way ANOVA followed by Dunnett's multiple comparisons test versus vehicle (raw data), *p*‐value: ns > 0.05, * ≤ 0.05, ** ≤ 0.01, *** ≤ 0.001, **** ≤ 0.0001.

The bioactivity of the rhizome, leaf, and fruit extracts was further evaluated using three models: intact PMNLs (functional cells), PMNL homogenates (lysed cells), and purified recombinant 5‐LOX. Overall, the extracts exhibited greater inhibitory potency against homogenates and the recombinant 5‐LOX than in intact cells (Figure 3a–c). This suggests that the active secondary metabolites have restricted access to their targets within intact cells, likely due to their high hydrophilicity and low lipophilicity, resulting in reduced membrane permeability. The leaf extracts evaluated against the purified 5‐LOX enzyme exhibited significant inhibitory activity, with the methanolic leaf extract demonstrating the greatest potency (Figure 3c). The concentration dependency was subsequently investigated for active extracts (Figure S1). The low IC_50_ values of the dichloromethane and methanolic rhizome and leaf extracts on PMNL homogenates and purified 5‐LOX reflect strong potency against leukotriene formation and suggest anti‐inflammatory action (Table 2). Importantly, none of the tested extracts reduced the viability of human primary monocytes in the MTT assay, supporting the specificity of their inhibitory activity on 5‐LOX without compromising cell viability (Figure 3d).

**TABLE 2:** IC_50_ values [µg/mL] for 5‐LOX product formation and the positive control zileuton in PMNL homogenate and the isolated enzyme 5‐LOX, mean with standard deviation, *n* = 3 [39].

	Rhizome extract			Leaf extract			Fruit extract			Zileuton
	DCM	MeOH	H_2_O	DCM	MeOH	H_2_O	DCM	MeOH	H_2_O	
**IC_50_ in PMNL homogenate**	3.4 ± 1.0	5.4 ± 2.4	> 30	2.2 ± 0.6	5.9 ± 1.6	> 30	> 30	> 30	> 30	0.2
**IC_50_ in 5‐LOX**	4.3 ± 1.5	11.8 ± 6.7	> 30	2.2 ± 1.6	1.5 ± 1.1	3.6 ± 2.4	> 30	22.3 ± 4.2	> 30	0.2

**FIGURE 4:**
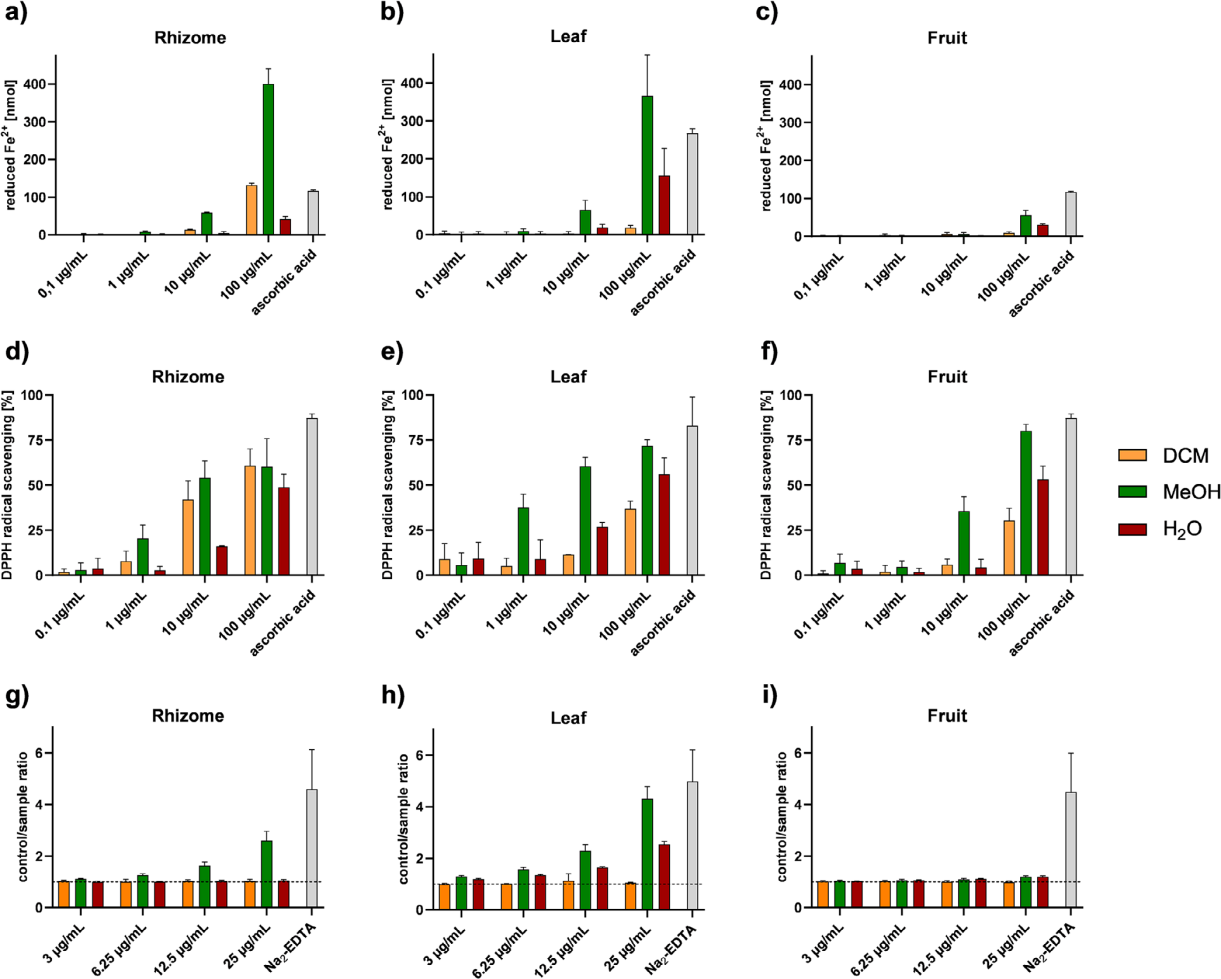
(a–c) FRAP assay: reduced ferrous (Fe^2+^) ions [nmol] by *R. chamaemorus* rhizome, leaf, and fruit extracts; ascorbic acid (8.8 µg/mL) as positive control; mean + standard deviation; *n* = 3‐4. (d–f) Percentual reduction of the free radical DPPH by *R. chamaemorus* rhizome, leaf, and fruit extracts; ascorbic acid (8.8 µg/mL) as reference; mean + standard deviation; *n* = 3‐4. (g–i) CAS assay on the iron complexing properties of *R. chamaemorus* rhizome, leaf, and fruit extracts; Na_2_‐EDTA (2.5 µg/mL) as positive control; measured absorption of control divided by absorption of samples; value is 1 in absence of samples; if substance binds iron, value increases; mean + standard deviation; *n* = 4‐5.

The catalytic domain of 5‐LOX contains a non‐heme iron in its active site. The iron plays a pivotal role in catalysis as an electron acceptor or donor. In the inactive state, the iron is in the form of Fe^2+^, while it is oxidized to Fe^3+^ for catalytic activity [40]. The only 5‐LOX inhibitor on the market, zileuton, acts at the catalytic domain by forming a complex with the iron in the active site [41]. Cloudberry leaves contain flavonoids, phenolic acids, and tannins, which may be responsible for the observed antioxidant and chelating properties (Figure 4) [42]. Based on this, it could be hypothesized that a combination of iron‐chelating and iron‐reducing properties may mediate the inhibition of 5‐LOX activity of the methanolic leaf extract (Figure 4a–c,g–i) [40, 42]. A significant reduction in 5‐LOX product formation was also observed with the dichloromethane extracts of the rhizome and leaves, as well as with the methanolic fruit extract. As there no chelating effect could be observed, this reduction must be explained by another mechanism (Figure 4g–i). The strong radical scavenging activity may contribute to the anti‐inflammatory effect (Figure 4d–f).

### 2.5 Extracts and HPLC Fingerprint Analysis

For a pre‐selection of the compounds from fruits, leaves, and rhizomes according to polarity, sequential extraction was performed using three solvents with increasing polarity. Of all plant parts, MeOH extraction resulted in the highest yield (28.5%–46.5%), whereas the DCM extracts showed the lowest yields (1.9%–6.7%) (Figure S2).

**FIGURE 5:**
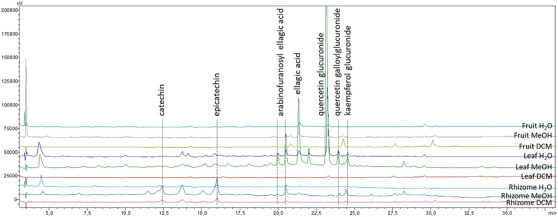
HPLC fingerprints of *R. chamaemorus* rhizome, leaf, and fruit extracts obtained with UV detector at 254 nm. Peak identification with MS via MRM.

The main compounds in the rhizome extract elute 8 min earlier than those in extracts of other plant parts (11–16.5 min vs. 19–25 min), suggesting a higher proportion of more polar compounds (Figure 5). The DCM rhizome extract shows only few small peaks. In particular, the methanolic rhizome extract contains high amounts of catechin (138.25 µg/mg extract) and epicatechin (128.89 µg/mg extract) (Table 3). The aqueous rhizome extract contains few UV–Vis active compounds. The fingerprints of all fruit extracts displayed few peaks, likely due to the predominance of sugars, which have weak UV absorption properties [43]. The dichloromethane leaf extract may contain polyunsaturated linolenic acid, pentacyclic triterpenic acids, carotenoids, and chlorophyll derivatives [42]. These compounds elute at the end of the method due to their low polarity, but remain poorly visible because of their weak UV absorption. The more polar methanolic and aqueous leaf extracts contain glycosylated derivatives of flavonoids, ellagic acids, catechin, and procyanidins [22, 23, 42]. The main peaks in the methanolic leaf extract were identified with mass spectrometry and quantified with HPLC: gallic acid (0.59 µg/mg extract), catechin (3.60 µg/mg extract), epicatechin (19.69 µg/mg extract), 4‐*O*‐α‐*L*‐arabinofuranosylellagic acid (11.31 µg/mg extract), ellagic acid (8.58 µg/mg extract), quercetin 3‐*O*‐β‐*D*‐glucuronide (94.80 µg/mg extract), quercetin 3‐*O*‐β‐*D*‐2‐galloylglucuronide, and kaempferol 3‐*O*‐β‐*D*‐glucuronide (5.24 µg/mg extract) (Table 3). Given the predominance of these polyphenols in the bioactive aqueous and methanolic leaf extracts, they may contribute to the observed antibiofilm and anti‐inflammatory activities. However, synergistic interactions between multiple compounds are also possible. The galloyl moiety in quercetin‐galloylglucuronide is associated with enhanced biological activity due to additional hydrogen bonding interactions [44]. Nevertheless, flavonoid glycosides often exhibit higher IC_50_ values for 5‐LOX inhibition, which may be attributed to reduced cellular uptake compared to their aglycone counterparts [40, 45].

**TABLE 3:** Content of compounds [µg/mg extract] in *R. chamaemorus* rhizome, leaf, and fruit extracts, quantified with HPLC, highest values in bold.

	Gallic acid	Catechin	Epicatechin	4‐*O*‐α‐*L*‐arabino‐furanosyl‐ellagic acid	Ellagic acid	Quercetin 3‐*O*‐β‐*D*‐glucuronide	Kaempferol 3‐*O*‐β‐*D*‐glucuronide
Rhizome DCM	0.26	33.53	31.96	1.17	0.87	2.06	0.16
Rhizome MeOH	0.21	**138.25**	**128.89**	4.64	1.39	2.06	3.04
Rhizome H_2_O	0.22	10.00	11.51	1.36	0.88	2.06	0.05
Leaf DCM	0.00	0.00	4.61	0.66	0.87	2.75	0.00
Leaf MeOH	0.59	3.60	19.69	**11.31**	**8.58**	**94.80**	**5.24**
Leaf H_2_O	0.56	1.97	1.75	2.53	1.50	29.80	1.32
Fruit DCM	0.00	0.55	0.28	0.00	0.91	0.00	0.00
Fruit MeOH	**0.96**	0.36	0.29	1.18	1.65	2.91	0.00
Fruit H_2_O	0.00	0.00	0.46	0.62	1.46	2.96	0.00

## 3 Conclusions

An overview of the biological activity of cloudberry fruits, leaves, and rhizomes was established. The focus was on their antibacterial, antifungal, cytotoxic, antibiofilm, and anti‐inflammatory properties, which are key factors in wound infections. In addition, the chelating, iron‐reducing, and antioxidant capacities were assessed to elucidate the potential mechanisms underlying the anti‐inflammatory effects.

Despite previously reported biological activities, the rhizome and particularly the fruit extracts showed only minor relevant effects in the applied assays. In contrast, the methanolic leaf extract containing nearly 10% (*w*/*w*) quercetin 3‐*O*‐β‐*D*‐glucuronide exhibited pronounced activity. Notably, it demonstrated strong biofilm‐inhibiting properties (MBIC of 30 µg/mL) and significant 5‐LOX inhibition (IC_50_ of 1.5 ± 0.5 µg/mL), indicating potential anti‐inflammatory activity. The observed biofilm inhibition may be related to interference with iron metabolism through extracellular iron chelation [46]. Further research is needed to determine whether the observed anti‐inflammatory activity could result from the chelation and reduction of the catalytically active iron. These combined properties could reduce persistent inflammation in chronic wounds and inhibit biofilm formation, which exacerbates the inflammation. The dichloromethane extracts of the rhizome and leaves also exhibited significant 5‐LOX inhibition (IC_50_ of 4.3 ± 1.5 and 2.2 ± 1.6 µg/mL, respectively). However, as they do not demonstrate relevant iron‐reducing and chelating properties, it appears that they interact with the enzyme differently than the methanolic leaf extract.

Furthermore, the absence of cytotoxic effects in HaCaT cells and monocytes, combined with the lack of direct antimicrobial activity against other ESKAPE pathogens and fungal strains, suggests the possibility of selective activity against specific bacterial targets. This mode of action could reduce the selective pressure for the development of resistance and minimize the risk of off‐target effects. Thus, the safety profile and suitability of the methanolic leaf extract for use in future topical drug formulations is enhanced. These observations provide a scientific basis for the traditional use of cloudberry leaves in wound healing. Additionally, they highlight the importance of investigating the whole plant and looking at individual plant organs separately, as their phytochemical composition and biological activities differ substantially.

Subsequent studies should prioritize bioactivity‐guided fractionation of the methanolic cloudberry leaf extract to isolate and characterize the compounds responsible for biofilm inhibition and 5‐LOX activity. Identifying these agents could pave the way for novel therapeutics targeting biofilm‐mediated wound infections.

By focusing on the leaf extraction, the yield of bioactive compounds is maximized while preserving rhizomes for plant regeneration and reserving fruits for nutritional or commercial purposes, thereby supporting both sustainability and resource optimization.

## 4 Experimental Section

### 4.1 Chemicals

(−)‐Epigallocatechin gallate (EGCG) (AA blocks, USA); trypsin (Biochrom GmbH; Germany); fetal bovine serum (FBS; Capricorn Scientific, Germany); A23187, arachidonic acid, prostaglandin B1 (PGB1), zileuton (Cayman Chemicals, USA); ellagic acid 4‐*O*‐α‐*L*‐arabinofuranoside (ChemFaces, China); GibcoTM RPMI 1640 medium, methanol (HPLC‐grade), anhydrous piperazine, trifluoracetic acid (Fisher Scientific, Germany); Dulbecco´s Buffer Substance (PBS) (SERVA Electrophoresis, Germany); dextrane 500, fetal calf serum (FCS), staurosporine, 2,2‐diphenyl‐1‐picrylhydrazyl (DPPH), trypane blue, 2,4,6‐Tris(2‐pyridyl)‐s‐triazine (TPTZ) (Merck KGaA, Germany); Mueller Hinton broth II, peptone from casein (pancreatic digest) (MerckMillipore, USA); gallic acid (Merck Schuchardt OHG, Germany); RPMI 1640 medium (PAN‐Biotec; Germany); quercetin‐3‐glucuronide (Phytolab, Germandy); chrome azurol S (CAS), etoposide, hexadecyltrimethylammonium bromide (HDTMA), 5‐sulfosalicylic acid (Sigma‐Aldrich, Germany); kaempferol‐3‐*O*‐glucuronide (TargetMol, USA); LymphoSep (Biowest – VWR, Germany); acetonitrile (LC‐MS grade), chloramphenicol, water (LC‐MS grade)(VWR International, USA); distilled water, dichloromethane, ethanol, methanol, phosphate‐buffered saline (PBS), and span agar (in‐house preparation, University of Greifswald). Unless mentioned otherwise, solvents and all other reagents were obtained from Carl Roth (Germany).

### 4.2 Plant Material

Leaves were harvested by A. A. Orlova in August 2019 in Lemblovo, Russia [23]. Fruits from *R. chamaemorus* were purchased online from Polarica in August 2024 (picked in Finland, Sweden) and stored at −20°C. Rhizomes were collected by Malte Zoerner and Dr. Kristina Kuprina in August 2024 in Kurkijärvi, Finland. All plant parts were identified by Prof. Dr. Martin Schnittler. Rhizomes and leaves were directly dried and stored with silica gel, while the fruits were lyophilized (Zirbus technology GmbH, Germany; RV8, Edwards, United Kingdom). Voucher specimens were deposited in the Herbarium of the University of Greifswald (Herbarium number: rhizome 060757; leaf 060758; fruit 060759). Prior to extraction, all plant materials were pulverized until obtention of a homogenous powder using a beater mill (A 11 basic, IKA‐Werke GmbH & Co. KG, Germany).

### 4.3 Bacterial and Fungal Strains

Most bacterial strains used belong to the ESKAPE pathogens [9]. The fungal species were selected from the WHO fungal priority pathogens list [47]. Detailed information on the strains is summarized in Table 4. The strains were stored in cryovials with a 20% glycerol solution at −80°C. Prior to use, the bacterial strains were streaked onto LB agar plates and incubated at 37°C for 24 h. A single colony was then picked and suspended in 5 mL of LB medium (Luria/Miller). Fungal strains were processed similarly, using Sabouraud‐dextrose (4% glucose) agar or medium instead of LB. The inoculum was incubated overnight at 37°C with shaking at 200 rpm.

**TABLE 4:** Characteristics of the pathogen strains used in this study. * 3/4MRGN classifies gram‐negative bacteria based on resistance to three or all of the following antibiotic groups: piperacillin as a penicillin derivative, cephalosporin with an extended spectrum, carbapenems, and fluoroquinolones [48].

Strain	Database numbers	Host	Origin	Resistances/type strain
*Acinetobacter baumannii*	DSM6974/PBIO2202	human	urine	intrinsic/type strain
	DSM9308/PBIO2212	human	endotracheal aspirate	intrinsic/type strain
*Enterococcus faecalis*	IMT4820/PBIO486	unknown	unknown	unknown
*Enterococcus faecium*	DSM13590/PBIO2713	human	feces, rectal swab, Germany	vancomycin‐resistant enterococci (VRE)
*Escherichia coli*	IMT17433/PBIO729	dog (*Canis lupus familiaris*)	urinary tract infection	extended spectrum beta‐lactamases (ESBL), 3MRGN*
	IMT16316/PBIO730	blackbird (*Turdus merula*)	feces	ESBL, 3MRGN*
	DSM1103/PBIO904	human	clinical isolate	EUCAST‐Ref. strain
*Klebsiella pneumoniae*	PBIO1990	human	tracheal secretion, clinical isolate, Germany	4MRGN*
	ATCC700603/PBIO2010	human	urine, USA	ESBL/EUCAST‐Ref. strain
*Pseudomonas aeruginosa*	PBIO712	fly	Rwanda	intrinsic
	DSM1117/PBIO2208	human	blood	intrinsic
*Staphylococcus aureus*	PBIO483	human	unknown	MRSA
	ATCC29213/PBIO901	human	wound	EUCAST‐Ref. strain
*Staphylococcus epidermidis*	ATCC 35984/PBIO4156	human	catheter sepsis, USA	unknown
*Streptococcus pyogenes*	PBIO4052	unknown	unknown	unknown
*Candida albicans*	DSM1386/PBIO837	human	bronchomycosis	unknown
	DSM11225/PBIO5430	human	blood, USA	unknown
*Aspergillus fumigatus*	IMT5874/PBIO3405	unknown	unknown	unknown
*Candida tropicalis*	PBIO5396	human	feeding tube, Germany	unknown
*Mucor circinelloides*	DMSZ 1175/PBIO3418	unknown	unknown	unknown

### 4.4 Extract Preparation

Powdered rhizomes, leaves, or fruits (500 mg) were mixed with 10 mL dichloromethane (DCM) and stirred for 1 h at room temperature to suspend the powder in the solvent. The mixture was then centrifuged at 1370 × g for 5 min, followed by decanting the supernatant, and the pellet resuspended in DCM. This process was repeated three times and the supernatants were combined. The extraction was subsequently performed with the remaining pellet using methanol (MeOH) and deionized water (H_2_O) as solvents, following the same procedure. The solvents were removed under reduced pressure at 40°C using a rotary evaporator (Rotavapor R‐114, Büchi, Switzerland; PC 3001 VARIOpro, Vacuubrand, Germany). Residual MeOH and DCM were evaporated at room temperature, while residual H_2_O in the aqueous extract was lyophilized (Zirbus technology GmbH, Germany; RV8, Edwards, United Kingdom).

### 4.5 Agar Diffusion Assay

Bacterial overnight culture in LB medium (150 µL) was plated onto Mueller–Hinton II (MHII) agar plates (25 mL per petri dish). 150 µL of fungal overnight culture in Sabouraud–dextrose broth containing 4% glucose (400 µL for PBIO3405 and PBIO3418) were plated onto Sabouraud–dextrose (4% glucose) agar plates (25 mL per petri dish). Disks (Ø 6 mm) loaded with 1 mg of plant extract were placed onto the agar. Negative controls consisted of disks containing only the solvent, while positive controls included disks loaded with 5 µg vancomycin hydrochloride, 10 µg ampicillin sodium salt, 10 or 30 µg gentamicin sulfate, 40 µg chloramphenicol, or 50 µg nystatin dihydrate (Table 5). After incubation for 24 h at 37°C, the antimicrobial activity was assessed by measuring the diameter of the inhibition zones (including the disk) using a ruler. All assays were conducted in biological triplicates.

**TABLE 5:** Positive controls of the fungal and bacterial strains.

Positive control	Strain	Strain number
5 µg vancomycin hydrochloride	*S. pyogenes, S. epidermidis*	PBIO4052, PBIO4156
10 µg ampicillin sodium salt	*E. faecium*	PBIO2713
10 µg gentamicin sulfate	*S. aureus, P. aeruginosa, E. coli, and A. baumannii*	PBIO483, PBIO712, PBIO901, PBIO904, PBIO2202, PBIO2208, PBIO2212
30 µg gentamicin sulfate	*E. faecalis*	PBIO486
40 µg chloramphenicol	*E. coli, K. pneumoniae, and C. albicans*	PBIO730, PBIO1990, PBIO2010, PBIO5340
50 µg nystatin dihydrate	*C. albicans, A. fumigatus, M. circinelloides, and C. tropicalis*	PBIO837, PBIO3405, PBIO3418, PBIO5396

### 4.6 Susceptibility Assay

20, 10 and 2 µL of a 10 mg/mL solution in MeOH of *R. chamaemorus* extracts were pipetted into the wells of a microtiter plate (96‐wells, sterile, Carl Roth, Germany). After allowing the solvent to evaporate, 198 µL of MHII medium was added to each well, followed by 2 µL of bacterial suspension with an OD_600_ of 0.5. Final extract concentration in the wells was 1, 0.5, and 0.1 mg/mL. Growth control, positive control with 50 µg/mL chloramphenicol (PBIO729, PBIO730) or 25 µg/mL gentamicin sulfate (PBIO483, PBIO901, and PBIO2202), respectively, 50 µg/mL gentamicin sulfate (PBIO2212), and blanks containing only extracts were included. Following 24 h of incubation at 37°C with shaking, the antimicrobial activity was visually determined. A clear solution indicates a growth inhibition. All tests were conducted in biological triplicates and technical duplicates.

### 4.7 Cell Viability Assay of Keratinocytes

#### 4.7.1 Cell Cultivation

Adherent human keratinocytes (HaCaT, RRID:CVCL_0038), CLS GmbH Germany, were cultured in RPMI 1640 medium supplemented with 8% FCS at 37°C and 5% CO_2_. Cells were subcultured twice a week. Therefore, medium was aspirated, monolayer washed with PBS/EDTA and then incubated with a trypsin/EDTA solution (0.05%/0.02% in PBS) for 9 min. The detached cells were centrifuged for 4 min by 123 × g and the pellet resuspended in fresh medium. Cell count was performed by the trypane blue exclusion method and the Luna^TM^ automated cell counter (Logos Biosystems; France). Cells were regularly tested for mycoplasma contamination.

#### 4.7.2 Determination of Cell Viability

24 h before the experiments, cells (1 × 10^4^ cells/well) were plated in a 96‐well plate in 100 µL of the medium and allowed to adhere. Stock solutions of the extracts were prepared in ethanol (EtOH) (for H_2_O extract, MeOH extract) or dimethyl sulfoxide (DMSO) (for DCM extract) and further diluted in medium (with final solvent concentration of 0.5% for EtOH or 0.2% for DMSO). Medium was aspirated, and 100 µL of each extract dilution was added to each well, and incubated for another 24 h. As a positive control, etoposide was used with a predetermined IC_50_ of 10 µM. Cells were treated with 100 µL thiazolyl blue tetrazolium bromide (MTT) at a final concentration of 0.5 mg/mL in growth media for 2 h in the incubator at 37°C and 5% CO_2_. The colored formazan crystals were dissolved by adding 100 µL of a lysis buffer (10% *w*/*v* SDS in 20 mM HCl). For complete dissolution of the formazan crystals, plates were incubated overnight. Absorbance was measured at 570 nm using a plate reader (FLUOstar Omega, BMG). The assay was performed as technical and biological triplicates.

### 4.8 Biofilm Inhibition Assay

**FIGURE 6:**
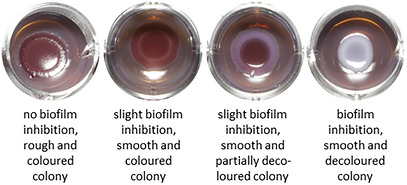
Inhibition of cellulose and curli fimbriae formation at varying intensities.

Various volumes (10, 20, 30, 40, 100, and 200 µL) of 1 mg/mL plant extract in MeOH were pipetted into the wells of a 24‐well plate (24‐wells, sterile, Sarstedt, Germany). After solvent evaporation, 1 mL of Congo red agar was added to each well (color solution: 50 mg Congo red, 25 mg Coomassie brilliant blue G250, 17.5 mL EtOH, 2.5 mL H_2_O.; 20 mL of the color solution was added to the agar: 15 g LB‐medium (Lennox), 18 g Span agar, 1 L deionized H_2_O). Subsequently, 5 µL of a bacterial suspension with an OD_600_ of 0.5 was applied on the agar. A sterile control, growth control and positive control with 50 µg/mL EGCG were included. Following 48 h of incubation at 27°C, the MBIC was assessed visually (Figure 6). All assays were performed in technical duplicates and biological triplicates. The protocol was adapted from Stepanov et al. [49].

### 4.9 Blood Cell Isolation

Human blood cells were freshly isolated from leukocyte concentrates derived from venous blood of healthy adult volunteers provided by the institute of Transfusion Medicine at the University Hospital Greifswald as described before [50, 51]. All methods were performed in accordance with the Declaration of Helsinki. Briefly, cells were immediately isolated by dextran sedimentation, followed by density gradient centrifugation on lymphocyte separation medium (LymphoSep ‐ Biowest, VWR, Darmstadt, Germany). Hypotonic lysis by water removed remaining erythrocytes, and resulting polymorph nuclear leukocytes (PMNL) were resuspended in PBS containing 0.1% (*w*/*v*) glucose (PG buffer) or PG buffer containing 1 mM CaCl_2_ (PGC buffer) as indicated. Peripheral blood mononuclear cells (PBMC) were seeded in RPMI 1640 (Merck) supplemented with 10% (*v*/*v*) heat‐inactivated FCS, 100 U/mL penicillin, 100 µg/mL streptomycin, and 2 mM L‐glutamine in cell culture flasks (Greiner Bio‐one, Frickenhausen, Germany) for 1.5 h at 37°C and 5% CO_2_ for isolation of human monocytes. Adherent monocytes were washed twice with PBS and were finally resuspended in RPMI 1640 medium as described before.

### 4.10 5‐LOX Product Formation in PMNL and Corresponding Homogenates

5 × 10^6^ PMNLs/mL were diluted in PGC buffer and pre‐incubated with extracts or controls (0.1% (*v*/*v*) vehicle) for 10 min at 37°C prior to stimulation with 2.5 µM A23187. After 10 min at 37°C, 5‐LOX product formation was stopped on ice and with one volume of ice‐cold MeOH. Afterwards, 530 µL acidified PBS and 200 ng of PGB_1_ as an internal standard were added, followed by purification using RP18 solid phase extraction. Formed 5‐LOX metabolites were eluted with MeOH and subsequently measured with RP‐HPLC using a C18 RP Radial PAK column (Waters, Eschborn, Germany) [52].

For the determination of 5‐LOX product formation in homogenates, 5 × 10^6^ PMNLs/mL were resuspended in PBS containing 1 mM EDTA. Cells were lysed on ice by 3 × 20 s sonication and resulting cell homogenates were pre‐incubated with extracts or vehicle (0.1%, *v*/*v*) for 10 min on ice. 5‐LOX product formation was started by the addition of 20 µM arachidonic acid and 2 mM CaCl_2_. After 10 min at 37°C, the reaction was stopped by the addition of ice‐cold MeOH, and 5‐LOX metabolites were analysed as described above for intact cells.

### 4.11 Expression and Purification of Human Recombinant 5‐LOX

*E. coli* (BL21) cells were transformed with the pT3‐5‐LO vector, and human recombinant 5‐LOX protein was expressed overnight at 30°C as previously described [51]. Cells were lysed in 50 mM Tris HCl pH 7.5, 200 mM NaCl, 5% (*v*/*v*) glycerol, 1 mM EDTA, 1 mM phenylmethylsulphonyl fluoride, 60 µg/mL soybean trypsin inhibitor, and 1 mg/mL lysozyme, homogenized by sonication (3 × 20 s), and centrifuged at 13000 × g for 45 min at 4°C. For purification of 5‐LOX, ATP affinity chromatography was used and the 13000 × g supernatant was loaded on an ATP‐agarose (Merck) column, washed with 50 mM phosphate buffer (PB) containing 1 mM EDTA, and eluted with PB supplemented with 1 mM EDTA and 20 mM ATP. Aliquots of semi‐purified 5‐LOX were diluted with PBS containing 1 mM EDTA and pre‐incubated with extracts or vehicle (0.1%, *v*/*v*) for 10 min at 4°C. Then, samples were stimulated with 2 mM CaCl_2_ and 20 µM arachidonic acid to start 5‐LOX product formation at 37°C. The reaction was stopped after 10 min by addition of ice‐cold MeOH, and the formed metabolites were analysed as described before for human primary blood cells.

### 4.12 Analysis of Cell Viability by MTT Assay

Monocytes (2 × 10^6^/mL in RPMI 1640 containing 10% heat‐inactivated FCS, 100 U/mL penicillin, 100 µg/mL streptomycin, and 2 mM L‐glutamine) were seeded in a 96‐well plate (100 µL/well). Cells were allowed to adhere for 1.5 h at 37°C, 5% CO_2_. Cells were incubated at 37°C and 5% CO_2_ with vehicle (0.5%, *v*/*v*) or compounds for 24 h. Then, cells were incubated with MTT (Merck KGaA, Germany, 5 mg/mL PBS) until blue staining of the vehicle‐containing control cells. Formazan formation was stopped by 100 µL SDS lysis buffer (10%, *w*/*v* in 20 mM HCl) followed by shaking overnight. Finally, absorbance was measured at 570 nm with a SpectraMax i3x multi‐mode detection platform (Molecular Devices, San Jose, CA, USA). The pan protein kinase inhibitor staurosporine (1 µM) was used as a cytotoxic control inhibitor [51].

### 4.13 CAS‐Assay

The assay was conducted based on the method described by Schwyn and Neilands, with modifications by Gerschler et al. [46, 53]. LC‐MS grade water was used throughout the assay. First, 6 mL of a 10 mM hexadecyltrimethylammonium bromide (CTAB) stock solution was mixed with 40 mL water. Separately, 1.5 mL of a FeCl_3_‐HCl stock solution (1 mM FeCl_3_ in 10 mM HCl) was combined with 7.5 mL of a 2 mM CAS dye solution. The Fe‐CAS mixture was then added to the CTAB solution under continuous stirring. To prepare the piperazine buffer, 6.5 mL of 12 N HCl was slowly added to 2 mL water, and 4.3 g anhydrous piperazine was completely dissolved. The piperazine acid solution was then slowly added to the Fe‐CAS‐CTAB solution, which was then brought to a final volume of 10 mL. Finally, 5‐sulfosalicylic acid was added to achieve a final concentration of 4 mM.

Stock solutions of 0.5 mg/mL were prepared. H_2_O and MeOH extracts were dissolved in water, and the DCM extract dissolved in DMSO. The stock solutions were diluted with water. Then, 100 µL of the CAS solution was added to each sample. The final extract concentrations were ranging from 3 to 25 µg/mL. After incubating for 1 h at room temperature in the dark, the absorption was measured at 630 nm (CLARIOstar Plus, BMG Labtech, Germany).

To account for the intrinsic absorption of the samples, a dilution series without CAS dye was measured and subtracted from the corresponding CAS‐containing samples. The controls consisted of water, the solvent of the extract, and the CAS solution. Iron‐chelation activity was expressed as the ratio of the absorbance of the control to the absorbance of the samples, where a value of 1 indicates no chelating activity. The assay was performed in three technical and four to five biological replicates.

$$\begin{array}{ccc} & & \mathrm{iron}\;\mathrm{chelation}\;\mathrm{activity}=\\ & & \frac{\mathrm{absorption}\left(\mathrm{control}\right)-\mathrm{absorption}\left(\mathrm{blank}\;\mathrm{of}\;\mathrm{control}\right)}{\mathrm{absorption}\left(\mathrm{sample}\right)-\mathrm{absorption}\left(\mathrm{blank}\;\mathrm{of}\;\mathrm{sample}\right)} \end{array}$$

### 4.14 DPPH Assay

The radical scavenger capability was investigated by measuring the reduction of the stable free radical DPPH. Briefly, an EtOH solution of a sample at various concentrations (extracts: 100 – 10 – 1 – 0.1 µg/mL) was mixed with an acetate‐buffered (pH 5–6.5) DPPH solution (50 µM, corresponding to 5 nmol). After incubation for 30 min in the dark, the absorbance of the mixture was measured at 520 nm with a SpectraMax i3x multi‐mode detection platform (Molecular Devices). Each extract was tested independently three times in duplicates. Extract absorbance values were normalized to vehicle controls. Ascorbic acid and L‐cysteine were used as reference compounds [54].

### 4.15 FRAP Assay

Antioxidant capacity was analysed by assessing the ability of extracts to reduce ferric ions (Fe^3+^) to ferrous ions (Fe^2+^) [55]. In brief, FRAP solution was prepared by mixing acetate buffer (10 mM, pH 3.6 by acetic acid) with ferric chloride hexahydrate (20 mM, dissolved in water) and 2,4,6‐Tris(2‐pyridyl)‐s‐triazine (TPTZ, 10 mM, dissolved in 40 mM HCl) at a ratio 10:1:1. In 96‐well plates, 20 µL vehicle (H_2_O), controls (ascorbic acid) or extracts (100 – 10 – 1 – 0.1 µg/mL) were incubated with 180 µL FRAP solution (pre‐warmed at 37°C and freshly prepared) for 5 min. Resulting absorbance was measured at 593 nm with a SpectraMax i3x multi‐mode detection platform (Molecular Devices). Each extract was tested independently three times in duplicates. Extract absorbance values were analysed by linear regression of a FeSO_4_ × 7 H_2_O concentration line.

### 4.16 Liquid Chromatography‐Mass Spectrometry Analysis

A high‐performance liquid chromatography (HPLC) method was developed for the detection of the main compounds. Analyses were performed on a Shimadzu chromatography system (LC‐20AD, Shimadzu Scientific Instruments, Japan) equipped with a photodiode array (PDA) detector and a LC‐MS‐8030 triple quadrupole mass spectrometer (Shimadzu Scientific Instruments, USA). As stationary phase served a Luna C18 column (100 Å, 250 × 4.6 mm) (Phenomenex, USA). The mobile phase consisted of LC‐MS grade H_2_O and acetonitrile (ACN) acidified with 0.1% acetic acid (Table 6). The flow rate was set at 1.2 mL/min and the column temperature at 45°C. The samples were dissolved in MeOH (1 mg/mL), the aqueous leaf and fruit extract in 50% (*v*/*v*) MeOH, filtered through a 0.22 µm nylon membrane filter (Carl Roth GmbH + Co. KG, Germany), and 20 µL were injected. UV detection was measured at 254 nm and 272 nm depending on the wavelength with higher absorption.

**TABLE 6:** Composition of the mobile phase (*v*/*v*).

Time (min)	ACN + 0,1% acetic acid (%)	H_2_O + 0,1% acetic acid (%)
0	5.0	95.0
15	16.0	84.0
35‐37	47.5	52.5
37.1‐40	5.0	95.0

This method was used to quantify the following compounds: gallic acid, catechin, epicatechin, 4‐*O*‐α‐*L*‐arabino‐furanosylellagic acid, ellagic acid, quercetin 3‐*O*‐β‐*D*‐glucuronide, and kaempferol 3‐*O*‐β‐*D*‐glucuronide. Calibration lines were determined five times using seven different concentrations. The lowest concentration was measured in triplicate (Table 7).

**TABLE 7:** Linearity of calibration for seven commercially available compounds in the cloudberry rhizome, leaf, and fruit extracts. Calibration equation: x = concentration [µg/mL], y = peak area [AUC].

Compound	Retention time (min)	Range (µg/mL)	R^2^	Calibration equation	Wavelength (nm)
Gallic acid	4.9	0.125–8	0.9993	*y* = 24816x‐2733	272
Catechin	12.6	0.500–32	1.0000	*y* = 4542x‐548	272
Epicatechin	16.2	0.500–32	0.9999	*y* = 6184‐376	272
4‐*O*‐α‐*L*‐arabino‐furanosylellagic acid	20.0 + 20.5	0.500–32	0.9990	*y* = 31656x‐17984	254
Ellagic acid	21.4	0.500–32	0.9991	*y* = 79766x‐66833	254
Quercetin 3‐*O*‐β‐*D*‐glucuronide	23.2	16–320	0.9998	*y* = 18329x‐36659	254
Kaempferol 3‐*O*‐β‐*D*‐glucuronide	24.6	0.500–32	0.9991	*y* = 16846x+5035	254

The mass spectrometer (Shimadzu Scientific Instruments, USA) was equipped with an electrospray ionization (ESI) source operated in positive and negative ion mode. Interface parameters were set as follows: nebulizing gas flow at 3 L/min, drying gas flow at 15 L/min, desolvation line temperature at 250°C, and heating block temperature at 400°C. Nitrogen was used as nebulizing gas, while argon served as collision gas.

The masses of 4‐*O*‐α‐*L*‐arabinofuranosylellagic acid, quercetin 3‐*O*‐β‐*D*‐glucuronide, quercetin 3‐*O*‐β‐*D*‐2‐galloylglucuronide, kaempferol 3‐*O*‐β‐*D*‐glucuronide and their fragments were monitored according the masses described by Whaley et al., followed by the comparison with pure compounds, the selection of the most intense precursor‐to‐product ion transitions and multiple reaction monitoring (MRM) optimization (Table 8) [22]. Additionally, MRM transitions were determined for gallic acid, catechin, epicatechin, and ellagic acid using pure compounds.

**TABLE 8:** MRM parameters for compounds detected in the rhizome, leaf, and fruit extracts at distinct retention times using a triple quadrupole mass spectrometer.

Compounds	Mode	MRM	Q1 pre‐Bias (V)	Collision energy (V)	Q3 pre‐Bias (V)
Gallic acid	ESI−	169.10 → 168.95	11	8	13
		169.10 → 125.00	11	17	17
		169.10 → 87.00	11	11	10
		169.10 → 79.05	11	25	11
Catechin	ESI+	291.15 → 291.05	−10	−6	−14
		291.15 → 139.00	−12	−19	−14
		291.15 → 123.10	−17	−15	−12
		291.15 → 164.90	−11	−14	−10
Epicatechin	ESI+	291.05 → 291.05	−10	−6	−14
		291.05 → 139.00	−11	−16	−30
		291.05 → 123.05	−11	−16	−12
		291.05 → 165.05	−10	−13	−16
4‐*O*‐α‐*L*‐arabino‐furanosylellagic acid	ESI+	435.00 → 434.90	−11	−7	−21
		435.00 → 302.95	−11	−20	−14
		435.00 → 257.05	−10	−49	−20
		435.00 → 275.00	−12	−41	−30
Ellagic acid	ESI−	301.00 → 300.90	13	7	23
		301.00 → 145.05	14	41	19
		301.00 → 228.80	14	26	17
		301.00 → 283.95	19	32	20
Quercetin 3‐*O*‐β‐*D*‐glucuronide	ESI+	478.85 → 303.05	−10	−16	−14
		478.85 → 479.05	−10	−7	−17
		478.85 →112.90	−10	−31	−26
		478.85 → 85.00	−10	−39	−20
Quercetin 3‐*O*‐β‐*D*‐2‐galloylglucuronide	ESI+	631.10 → 631.05	−24.0	−5.0	−22.0
		631.10 → 329.00	−24.0	−16.0	−24.0
		631.10 → 302.95	−24.0	−25.0	−21.0
Kaempferol 3‐*O*‐β‐*D*‐glucuronide	ESI +	462.85 → 286.90	−14	−18	−13
		462.85 → 463.05	−15	−6	−22
		462.85 → 112.95	−14	−30	−27
		462.85 → 85.10	−15	−42	−19

### 4.17 Software

Shimadzu LabSolutions version 5.85 was used for LC and MS data analysis. GraphPad Prism version 8.0.2 and 10.2.2 were employed for the creation of the graphs and R version 4.4.2 for the statistical analysis.

## Author Contributions

**Hildegard Kieninger**: writing – review and editing, writing – original draft, visualization, investigation, data curation, and formal analysis. **Stefanie Koenig**: writing – original draft, writing – review and editing, investigation, and data curation. **Nadin Schultze**: writing – original draft, writing – review and editing, visualization, investigation, data curation, and conceptualization. **Ulrike Garscha**: writing – review and editing, supervision, and project administration. **Sebastian Guenther**: writing – review and editing, supervision, project administration, funding acquisition, and conceptualization. **Christian Schulze**: writing – review and editing, investigation, supervision, project administration, funding acquisition, and conceptualization.

## Funding

Most of the research was funded by the German Federal Ministry for Food and Agriculture (BMEL) through the Fachagentur Nachwachsende Rohstoffe e. V. (FNR), grant number 2221MT012X. The section covering the anti‐inflammatory, DPPH and FRAP assay received no external funding (Greifswald).

## Conflicts of Interest

The authors declare no conflicts of interest.

## Institutional Review Board Statement

The study was conducted by the Declaration of Helsinki and approved by the Institutional Review Board (or Ethics Committee) of University medicine Greifswald (protocol code BB 014/14, approved 11.02.2014) for studies involving humans. After requesting clarification on the applicability of Nagoya rules in Sweden, the Swedish Environmental Protection Agency informed us that Sweden has not implemented national legislation under the Nagoya Protocol or ABS; thus, the species intended for collection do not fall under the EU ABS Regulation and no PIC or MAT are required. Correspondingly, the Finnish Environment Institute (SYKE) confirmed that no national Nagoya/ABS legislation applied to the studied species in Finland.

## Supporting information




**Supporting File 1**: cbdv71479‐sup‐0001‐SuppMat.docx.

## Data Availability

The data that support the findings of this study are available from the corresponding author upon reasonable request.
