# Supplementary material for: Antibiofilm, Antimicrobial, and Anti‐inflammatory Potential of Rubus chamaemorus Rhizome, Leaf, and Fruit Extracts
Source: Chem Biodivers. 2026 Jul 9;23(7):e71479. doi: 10.1002/cbdv.71479 (PMC13348868; doi:10.1002/cbdv.71479)
Supplement: Supplementary file 1 — Supporting File 1: cbdv71479‐sup‐0001‐SuppMat.docx. [file CBDV-23-e71479-s001.docx]

**Supplementary data**

**Figure S1: a), b), c)** Concentration-dependent inhibition of 5-LOX product formation of PMNL homogenates after DCM, MeOH, and H_2_O rhizome, leaf or fruit extract treatment; mean ± standard deviation; n=3-4. **d), e), f)** Concentration-dependent inhibition of 5-LOX product formation of purified recombinant 5-LOX after DCM, MeOH, and H_2_O rhizome, leaf or fruit extract treatment; mean ± standard deviation; n=3.

**Figure S2**: Yield of extraction, mean + standard deviation in % of dry mass, n=3-14.
